# Congenital etiologies of exocrine pancreatic insufficiency

**DOI:** 10.3389/fped.2022.909925

**Published:** 2022-07-22

**Authors:** Isabelle Scheers, Silvia Berardis

**Affiliations:** ^1^Department of Pediatrics, Pediatric Gastroenterology and Hepatology Unit, Cliniques Universitaires Saint Luc, Université Catholique de Louvain, Brussels, Belgium; ^2^Department of Pediatrics, Specialized Pediatrics, Pediatric Pneumology and Cystic Fibrosis Unit, Cliniques Universitaires Saint Luc, Université Catholique de Louvain, Brussels, Belgium

**Keywords:** exocrine pancreatic insufficiency, cystic fibrosis, Shwachman-Bodian-Diamond syndrome, Pearson syndrome, Johanson-Blizzard syndrome, pancreas agenesis

## Abstract

Congenital exocrine pancreatic insufficiency is a rare condition. In a vast majority of patients, exocrine dysfunction occurs as part of a multisystemic disease, the most prevalent being cystic fibrosis and Shwachman-Bodian-Diamond syndrome. Recent fundamental studies have increased our understanding of the pathophysiology of these diseases. Exocrine pancreatic dysfunction should be considered in children with failure to thrive and fatty stools. Treatment is mainly supportive and consists of pancreatic enzyme replacement and liposoluble vitamins supplementation.

## Introduction

The pancreas is a mixed endocrine and exocrine gland. The exocrine tissue comprises acinar cells that produce and store pancreatic enzymes; and ductal cells that secrete fluid and electrolytes. Digestive pro-enzymes flow into the duodenal lumen where they are activated and ensure nutrient digestion. The exocrine pancreatic function is immature at birth (1–3). Pancreatic amylase activity is nearly absent in premature and term neonates, remains low in the first year of life to progressively reach adult values around 3 years of age. Trypsin and lipase activity are markedly lower (10x and 20x, respectively) at birth but progressively reach adult values within the first year of life. The functional exocrine reserve is impressively large as more than 98% of the pancreatic enzyme production must be lost before steatorrhea develops (4, 5). Exocrine pancreatic insufficiency (EPI) is usually evidenced in infants with clinical features of malabsorption such as failure to thrive, chronic diarrhea, anemia or hypoalbuminemia. Fecal elastase-1 is the most frequent test used to identify pancreatic insufficient (PI) patients.

Although a wide variety of conditions may be associated with EPI, most are syndromic and exceedingly rare. Etiologies of congenital EPI can be subdivided in 3 groups based on underlying pathophysiologic mechanisms: i.e., related to (a) exocrine pancreatic tissue injury, (b) pancreatic hypoplasia or agenesis or (c) isolated enzyme deficiency. Cystic fibrosis (CF) represents by far the most frequent cause of inherited EPI (90–95%) followed by Shwachman-Bodian-Diamond syndrome (SBDS, ~4%). The differential diagnosis and main cardinal features are summarized in Table 1.

**Table 1:** Etiologies and main clinical features of congenital exocrine pancreatic insufficiency.

	**Genetic defect**	**Main clinical features**
**Exocrine pancreas tissue injury**		
Cystic fibrosis	*CFTR*	Chronic sinopulmonary disease (e.g.,) -Chronic cough and sputum production -Chronic wheezing and air trapping -Obstructive lung disease -Nasal polyps -Chronic pansinusitis Digestive system (e.g.,) -EPI or pancreatitis -Meconium ileus -Liver disease (cholestasis, steatosis, portal HT) -Failure to thrive -Distal intestinal obstruction Obstructive male infertility -Hypoplasia, aplasia of vas deferens -Hypoplasia, aplasia of seminal vesicles Other -Diabetes mellitus
Shwachman-Bodian-Diamond syndrome type 1	*SBDS*	Hematologic anomalies related to bone marrow dysfunction (e.g.,) -Hypoproductive cytopenia -Pancytopenia -Leukemia EPI Skeletal dysplasia -Short stature -Thoracic dystrophy -Chondrodysplasia Other -Hearing loss, ear malformation -Cardiac defects -Increased liver enzymes, hepatomegaly -Delayed teeth eruption, dysplastic teeth -Kidney tubulopathy -Psychomotor delay -Ichthyosis -Eye anomalies (strabismus, coloboma, keratitis)
Shwachman-Bodian-Diamond syndrome type 2	*EFL1*	(See SBDS type 1) Specific features -Myopia -Arched palate
SBDS-like	*eIF6*	(See SBDS type 1)
SBDS-like	*DNAJC21*	(See SBDS type 1) Specific features -Retinal dystrophy -Hypermobile joints
		-Hip dysplasia -Cryptorchidism
SBDS-like	*SRP54*	(See SBDS type 1) Specific features -Congenital profound neutropenia
Pearson syndrome	mt-Deletion	Hematologic anomalies -Sideroblastic anemia -Vacuolization of marrow precursors Digestive system -EPI -Hepatomegaly, increased liver enzymes Other -Muscle weakness (ptosis, limb weakness) -Endocrinologic disturbance (hypothyroidy, hypoparathyroidy, growth hormone deficiency, adrenal Insufficiency, diabetes mellitus) -Splenic atrophy -Impaired cardiac function -Renal insufficiency -Cardiac conduction block
Johanson Blizzard syndrome	*UBR1*	Nasal alea hypoplasia/agenesis EPI Mental or psychomotor retardation Other -Short stature -Scalp defects, alopecia, abnormal hair implantation -Oligodontia, microdontia -Imperforate anus -Genito-urinary anomalies (vesico-ureteral reflux hypospadias) -Endocrinologic disturbance (hypothyroidy, hypopituitarism, diabetes mellitus)
Shteyer syndrome	*COX4I2*	Dyserythropoietic anemia EPI Calvarial hyperostosis Other -Delayed psychomotor development -Hepatomegaly -Splenomegaly -Muscle weakness -Dental anomalies (maldentition, caries)
Pancreas hypoplasia/agenesis	*PDX1*	Neonatal diabetes mellitus
		EPI
	*PTF1A*	Neonatal diabetes mellitus EPI Cerebellar agenesis/hypoplasia Other -Optic nerve hypoplasia -Joint stiffness -Dysmorphic facial features (triangular face, beaked nose, low set dysplastic ears) -Little subcutaneous fat.
	*GATA6*	Congenital heart disease Neonatal diabetes mellitus EPI Developmental delay Other -Bile tract anomalies (biliary atresia, gallbladder agenesis) -Endocrine anomalies -Diaphragmatic hernia
Isolated enzyme deficiency	*PNLIP*	Steatorrhea

*EPI, exocrine pancreatic insufficiency; HT, hypertension; SBDS, Shwachman-Bodian-Diamond syndrome*.

The purpose of the present mini-review is to depict the differential diagnosis in EPI and summarize advancing knowledge on the pathophysiologic mechanisms leading to EPI in those conditions.

## Congenital exocrine pancreatic insufficiency due to exocrine injury

### Cystic fibrosis

**Figure 1:**
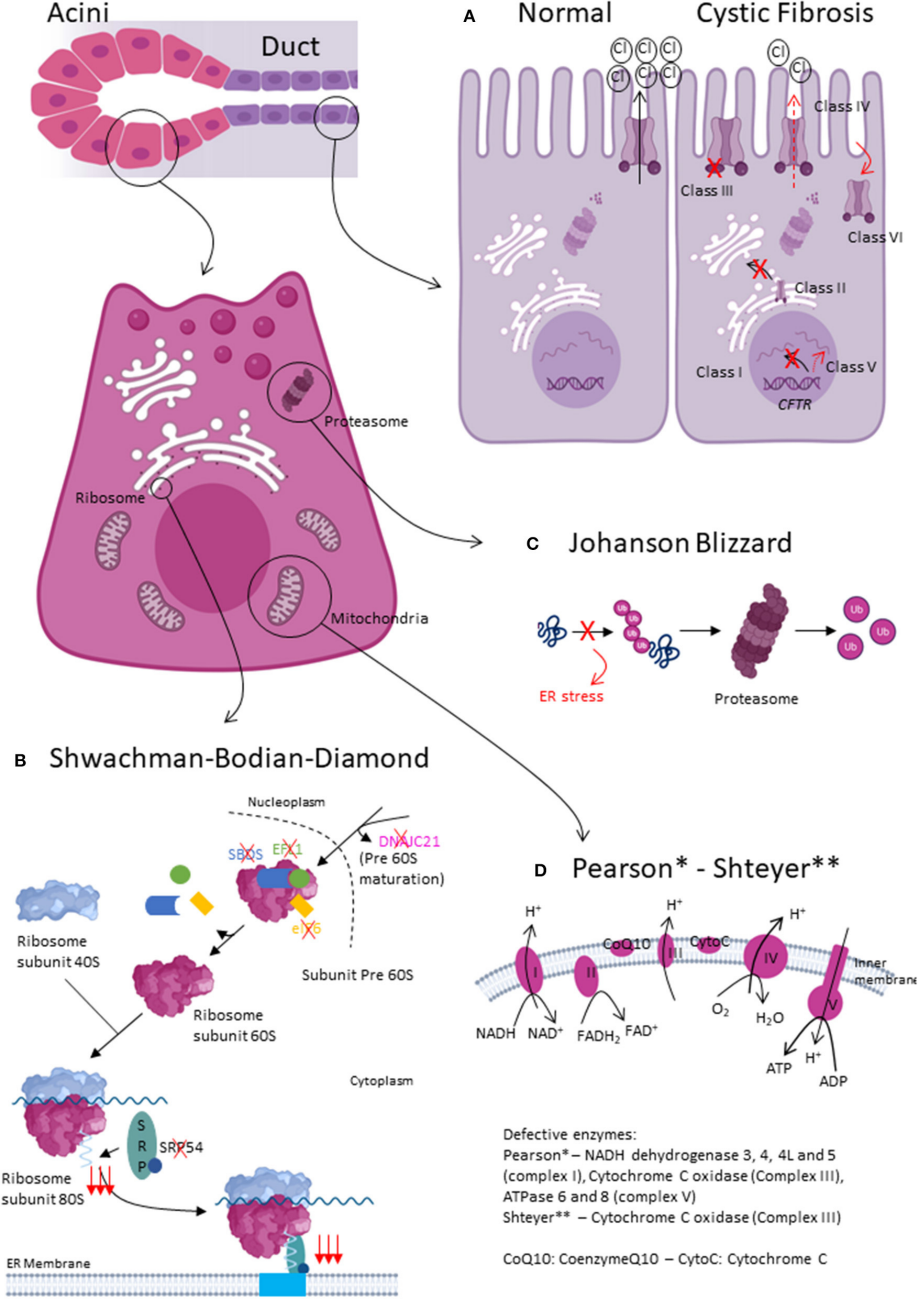
Pathophysiology of exocrine pancreatic insufficiency due to pancreatic exocrine tissue injury. **(A)** Normal and altered CFTR function. In Class I *CFTR* mutations, no CFTR protein is synthetized. In Class II, CFTR protein trafficking is defective. Class III mutations lead to impaired gating, whereas Class IV lead to impaired conductance. In Class V and VI, there is, respectively, less CFTR protein or the protein is less stable. **(B)** Shwachman-Bodian-Diamond type1, type2 and SBDS-like syndromes mainly impact acinar cell function. All disease-causing mutations impact the final maturation steps of ribosome biogenesis. **(C)** Johanson Blizzard syndrome mainly effects acinar cell function. UBR1 mutations cause defective recognition of misfolded proteins, which can therefore not be degraded by the proteasome. **(D)** Pearson and Shteyer syndrome lead to mitochondrial dysfunction. Acini seem more affected than ductal cells.

Cystic fibrosis (CF) is an autosomal recessive, multisystemic disorder caused by mutations in the cystic fibrosis transmembrane conductance regulator (*CFTR*) gene. CF has an estimated prevalence of 1/3.000 in Caucasians. The CFTR gene codes for a cAMP-responsive chloride channel at the apical surface of secreting epithelia (6, 7). More than 2000 *CFTR* mutations were described to date. The most frequent, occurring at least on 1 allele in >65% CF patients, is a 3-base-pair deletion causing the loss of a phenylalanine at position 508 of the protein (F508del). *CFTR* mutations have been classified in 6 classes based on their predominant effect on CFTR function or processing (Figure 1A) (8, 9).

Pancreatic damage in CF already begins in utero (10). As from 32 weeks of gestation, the acinar and duct lumen were found to be dilated by inspissation of proteinaceous material. This duct plugging causes intrapancreatic enzyme activation, gland inflammation and leads to acinar injury; a process that progresses through infancy until the exocrine tissue is replaced by fibrotic cells. Initially, endocrine cells are relatively preserved. Functional testing confirms reduced fluid, and subsequently, enzyme secretion in CF patients (11). In pancreatic sufficient (PS) children, this process is delayed as mutations have a milder functional impact on CFTR.

CF is diagnosed in many countries by newborn screening evidencing high serum trypsinogen levels. Later in life, CF patients present with chronic cough, rectal prolapse, steatorrhea, failure to thrive or male infertility. The final diagnosis of CF is based on sweat-test and *CFTR* sequencing (12). Transepithelial nasal potential difference may help to classify patients with mutations of unknown functional impact. Most clinical symptoms of CF arise from the dehydration of sweat, mucus or digestive fluids. The CFTR defective gene causes the secretions to become sticky and thick. Instead of acting as lubricants, the secretions clog up tubes and ducts, especially in the lungs, gut and pancreas. Cardinal features are summarized in Table 1. CF patients with identical CFTR genotypes show a high variability in disease severity, complication rates and survival. These differences are largely attributable to genetic modifiers (variants in other genes such as *SLC26A9 or TGFB1*) and environmental factors (such as cigarette smoke exposure or bacterial pathogens).

Cross sectional studies of CF cohorts evidenced that almost 85% of patients present EPI (13, 14). Before the advent of CFTR modulators, CF children carrying two class I (reduced or absent synthesis) or II (block in protein processing) mutations became pancreatic insufficient (PI) within the first year of life (15).

EPI management encompasses nutritional support, pancreatic enzyme replacement therapy (PERT) and liposoluble vitamin supplementation following existing guidelines (16). Recent major therapeutic advances in CF care concern a more fundamental and targeted treatment of the disease, using CFTR modulators. CFTR modulators are small molecules which act specifically at the level of the defective mechanisms causing CF. The effects of these modulators on exocrine pancreatic function have not yet been studied as primary outcomes but rather as secondary or exploratory outcomes. Nevertheless, several data suggest that CFTR modulators may at least partially restore the exocrine pancreatic function in CF patients. Fecal elastase-1 rose above the clinical cutoff of 200 μg/g at least once in >25% of CF children with a CFTR gating-mutation treated with Ivacaftor for 24 weeks (17). Ivacaftor is a CFTR modulator that increases the open probability of CFTR, leading to enhanced chloride transport. Moreover, a reduction in immunoreactive trypsinogen (IRT) concentrations, a marker of pancreatic stress, has also been observed. Another study showed that the increase of fecal eslastase-1 and the decrease of serum IRT were maintained for an additional 84 weeks in CF children with a CFTR gating-mutation treated with Ivacaftor, suggesting that early CFTR modulation can potentially delay the decline of pancreatic function (18). The same tendency has been observed in CF children homozygous for F508del and treated with Ivacaftor/Lumacaftor combination therapy for 24 weeks (19). The effect of the triple combination (Elexacaftor/Tezacaftor/Ivacaftor) on the exocrine pancreatic function has not been studied yet. However, a significant increase in BMI in CF patients (heterozygous carriers for F508del and a minimal function CFTR mutation as well as in CF patients homozygous for F508del-CFTR mutation) treated with this medication has been described. The effect of CFTR modulators on BMI can be explained by different factors including a decreased resting energy expenditure, decreased gut inflammation, increased exocrine pancreatic function and an increased appetite (20–22). Other authors observed similar effects on growth parameters (23–25). Additional research is needed to further understand the specific effect of these modulators on exocrine pancreatic function.

Long-term survival of patients with CF is mainly dictated by the decline in lung function and by the occurrence of cirrhosis (8). Over the past decades, earlier diagnosis and careful disease management led to a dramatic improvement of patient quality of life and life expectancy which may now reach 50 years (26).

### Shwachman-Bodian-Diamond syndrome type 1, type 2 and SBDS-like syndromes

Shwachman-Bodian-Diamond syndrome (SBDS) is the second most frequent cause of congenital EPI. Although originally described by Nezelof in 1961, the disease was named after physicians Shwachman, Bodian and Diamond in 1964. SBDS prevalence is estimated at 1/76.000 (27).

Approximately 90% of SBDS patients carry biallelic mutations in the Shwachman-Bodian-Diamond syndrome gene (*SBDS*, SBDS type1). More recently, the molecular spectrum of SBDS has been extended with biallelic mutations evidenced in elongation factor-like GTPase 1 (*EFL1*, SBDS type2), DnaJ heat shock protein family member C21 (*DNAJC21*), eukaryotic initiation factor (*eIF6*) or heterozygous mutation in signal recognition particle 54 (*SRP54*) genes. Although SBDS might seem genetically heterogeneous, all encoded dysfunctional proteins affect the final maturation steps of the large ribosomal subunit (Figure 1B). Ribosome biogenesis is a highly conserved and tightly regulated process involving more than 200 ribosomal proteins, ribosomal RNAs and small nuclear RNA molecules. Ribosome synthesis takes place in the cytoplasm and nucleolus. DNAJC21 seems involved in the biogenesis of nucleolar rRNA and in the cytoplasmic recycling of a factor involved in the nuclear export of the 60S ribosomal subunit. Furthermore, SBDS cooperates with EFL1 to release eIF6, an anti-association factor, from the 60S ribosomal subunit; enabling the 40S and 60S subunits to assemble and form the 80S ribosome. Finally, SRP54 is part of the SRP complex that escorts the nascent polypeptide of the 80S ribosome to the endoplasmic reticulum (ER) (28).

Pancreas necropsy in SBDS patients evidenced acinar hypoplasia and extensive fat infiltration without fibrosis or inflammation; the ductal cells and Langerhans islets were conserved. In parallel, quantitative pancreatic function tests in SBDS patients showed, in relation to the total acinar secretion, normal fluid and anion outputs but severely reduced enzyme secretion (29). Conditional *SBDS* KO and hypomorphic mutants phenocopied SBDS in mice. Progressive acinar cell atrophy and reduction in number and size of zymogen granules, organelles storing pancreatic enzymes in acinar cells, were evidenced as from the post-natal period. These pathologic findings correlate with reduced pancreatic enzyme production. Acinar depletion was shown to result from p53 dependent senescence cell cycle arrest. However, the precise mechanism by which impaired ribosome biogenesis activates p53 remains unclear (30, 31).

A vast majority of patients with biallelic SBDS mutations are diagnosed during infancy with more or less severe infections and/or signs of malabsorption and failure to thrive (90%). Pancreatic ultrasound may show fatty gland changes.

The cardinal features of SBDS are bone marrow failure, EPI and skeletal anomalies (Table 1).

The management of EPI in SBDS patients is supportive and relies on PERT and liposoluble vitamin supplementation. A modest improvement in exocrine function can be seen over time in about 45% of patients to an extent they could discontinue PERT (32, 33). Reasons for this improvement remain obscure, although it is believed to be related to the physiologic maturation of pancreatic enzyme secretion with age. Regular pancreatic function monitoring is therefore warranted to diagnose patients that converted from PI to PS status. Serum trypsinogen has been shown to be a reliable marker to follow exocrine function in SBDS patients (34). Trypsinogen concentration below 50 μg/L (normal values 140–400 μg/L) are seen in PI patients and rise above 50 μg/L in PS patients, sometimes reaching normal values.

Long-term survival in SBDS patients is primarily dictated by severe infections and hematologic malignancies. Early development (24 and 38 years) (35, 36) of pancreatic cancer was also described in SBDS type1. Survival at 20 years was 87.4% (95%CI 75.3–93.8) in an Italian SBDS cohort (37) but remains poorly investigated beyond that age.

### Johanson-Blizzard syndrome

Johanson-Blizzard syndrome (JBS) is a multisystemic autosomal recessive disorder with a prevalence estimated at 1/250.000 (38). JBS was first described in 1967 by Morris and Fisher (39), but was ultimately named after Drs Johanson and Blizzard in 1971 who delineated the syndromic spectrum of the disease (40). JBS was later found to be caused by homozygous or compound heterozygous mutations in the ubiquitin-protein ligase E3 component N-recognin 1 (*UBR1*) gene encoding one of a handful ligases of the N-end rule pathway (38). As such, wild-type UBR1 recognizes, binds and mark N-terminal residue of proteins ultimately leading to protein degradation by the proteasome. Mutated *UBR1* is hypothesized to interfere with proper protein degradation and result in unfolded protein accumulation in the ER, causing ER stress (Figure 1C).

Pancreas necropsy in JBS patients evidenced progressive acinar tissue loss and inflammatory infiltrates (41). This pathogenic process was shown to start during fetal life. Functional tests in pancreas of UBR1^−/−^ mice showed markedly decreased zymogen outputs compared to controls following cholecystokinin injection, as well as increased susceptibility to pancreatitis. These findings suggest that the N-end rule pathway may be involved in zymogen processing or export (38) and that defective zymogen trafficking play a role in pancreatitis. Similarly, quantitative pancreatic function tests in JBS patients showed significantly decreased enzyme secretion but preserved fluid and anion outputs (29). Furthermore, serum trypsinogen concentration were markedly below normal ranges.

The cardinal features of JBS are EPI and hypo- or aplasia of the nasal wings (Table 1). EPI is invariably diagnosed during early infancy and doesn't improve over time. As *UBR1* expression is ubiquitous, all organs may be affected. Other facultative features include cranio-facial anomalies (dentition anomalies, scalp defects, microcephaly, cleft palate), short stature, developmental delay, congenital heart disease, endocrine glands dysfunction (hypothyroidism, diabetes mellitus), raised liver enzymes, genito-urinary and kidney defects.

The management of EPI in JBS patients include PERT and liposoluble vitamin supplementation. Endocrine hormone supplementation may further be required.

With adequate treatment, JBS patients survive into adulthood.

### Pearson syndrome

Pearson syndrome is a very rare mitochondrial cytopathy, with an estimated prevalence <1/1.000.000. The disease was first described by Pearson in 1979 (42).

Rotig et al. discovered in 1995 that Pearson syndrome was caused by deletions ranging from 1.1-10kb in mitochondrial DNA (mtDNA) (43). Deletions give rise to 3 overlapping phenotypes: Pearson syndrome, Kearns Sayre and progressive external ophtalmoplegia. The disease expressivity doesn't seem related to the size or location of mtDNA deletion but rather to heteroplasmy (i.e., relative abundance of mitochondria carrying the mutation in each cell), tissue distribution (random partitioning of mitochondria) and tissue-specific vulnerability to oxidative stress. In Pearson syndrome, the deletion was found to be more abundant in blood compared to other cells. MtDNA encodes amongst others ATPases 6 and 8, cytochrome c oxidase III and NADH dehydrogenase 3, 4, 4L and 5. Hence, MtDNA deletion results in defective oxidative phosphorylation and impaired translation of messenger RNAs to proteins (Figure 1D). Pearson syndrome occurs sporadically which is suggestive for mutations arising de novo during either oogenesis or early embryonic development.

Histopathology of the patients' pancreas is characterized by acinar cell loss which are replaced by connective tissue and blood vessels. Ductal cells and Langerhans islets seem largely unaltered. However, functional pancreatic tests show not only decreased acinar function but also impaired fluid and electrolyte secretion (42).

Cardinal clinical features of Pearson syndrome are refractory sideroblastic anemia, bone marrow precursors vacuolization and EPI (Table 1). Patients with Pearson syndrome invariably develop EPI during infancy. The diagnosis is further comforted by increased serum lactate/pyruvate and ketone body ratios (44).

Patient management is mainly based on the symptomatic treatment of EPI (PERT and liposoluble vitamins supplementation), pancytopenia (folic acid, transfusion) and, although not evidenced-based, some authors have suggested to support the mitochondrial electron transport chain (L-carnitine, coenzyme Q). Of note, hypercaloric diets, high glucose containing diets and parenteral nutrition might precipitate mitochondrial dysfunction (44–46).

Pearson syndrome is often fatal in early childhood. Patient surviving this period develop symptoms of Kearns Sayre Syndrome; a neuromuscular disease characterized by early onset ophtalmoplegia and pigmentary retinopathy.

### Shteyer syndrome

Shteyer syndrome is an exceptional multisystemic autosomal recessive disorder caused by biallelic mutations in the *COX4I2* gene coding for a component of the cytochrome c oxidase, the terminal enzyme in the mitochondrial respiratory chain (Figure 1D).

Cardinal disease features are exocrine pancreatic insufficiency, dyserythropoietic anemia and calvarial hyperostosis (Table 1). To date 4 patients have been described with the disease (47), all presenting failure to thrive and steatorrhea soon after birth. On imaging, the pancreas appeared atrophic and fatty.

The disease management is mainly based on the symptomatic treatment of EPI (pancreatic enzyme and liposoluble vitamins supplementation) and iterative transfusions for anemia.

Follow-up data is lacking to determine the outcome of affected patients.

## Congenital pancreatic hypoplasia or agenesis

A handful patients present isolated or syndromic pancreas agenesis and subsequent exocrine and endocrine pancreatic insufficiency related to mutations in genes coding for transcription factors playing a critical role in early cell fate and pancreas development.

### Isolated pancreas hypoplasia/agenesis

Pancreas agenesis is caused by biallelic mutations in *PDX1* gene (48), a transcription factor critical for pancreas development (49). Heterozygous patients develop maturity onset diabetes of the young type 4 (MODY4).

### Syndromic pancreatic agenesia and congenital heart defects

The syndrome is caused by heterozygous mutations in *GATA6*, an important zinc-finger transcriptional regulator in the development and differentiation of numerous tissues (50). It has been suggested that heterozygous *GATA6* mutations result in protein loss of function and cause pancreatic agenesis through haploinsufficiency. Less than 40 patients were reported to date and most patient harbored *de novo* mutations (50–52).

The patient phenotype associates pancreas hypoplasia or agenesis, cardiac defects (ventricular septal defects, atrial septal defects, pulmonary stenosis, tetralogy of Fallot) and developmental delay (Table 1).

### Syndromic pancreatic and cerebellar agenesis

Pancreas and cerebellar agenesis syndrome is caused by biallelic mutations in *PTF1A* gene encoding a transcription factor involved in cell fate during early pancreas development (53). The disease was described to date in 6 individuals (53–56), all born from consanguineous parents.

The patient's phenotype mainly associates EPI, neonatal diabetes mellitus and cerebellum agenesis (Table 1).

Patient life expectancy is short (<6 months).

## Congenital isolated enzyme deficiency

Congenital pancreatic lipase deficiency is an exceptional mono-enzymatic cause of EPI. The disease results from by biallelic mutations in *PNLIP* gene (57). Only a handful patients have been described with the disease world-wide.

Dietary fat digestion relies on the joint action of pancreatic lipase, colipase and bile salts. In the duodenum, triglycerides are emulsified by bile salts. Colipase enables lipase to anchor the surface of lipid micelles and hydrolyze dietary long chain triglycerides to free fatty acids and monoacylglycerols. The main clinical symptom is steatorrhea and treatment relies on PERT.

Patients with isolated colipase (*CLPS* gene) (58) or combined lipase-colipase (59, 60) deficiency have historically been described but none were confirmed genetically.

## Summary and perspectives

This mini-review illustrates the remarkable complexity and diversity of pathways leading to inherited EPI. The improvement of genetics has allowed to define the etiology of congenital EPI in a majority of patients. These progresses have led to a better understanding of the relationship between those gene mutations and the pathophysiologic mechanisms leading to altered pancreatic function. Until a decade ago, the treatment of EPI was essentially symptomatic. Since then, the discovery of targeted treatments in cystic fibrosis raises many hopes to slow down the exocrine function decline. The development of specific treatments for other rare causes of EPI remains a great challenge for the future.

## Author contributions

SB drafted the CF part of the manuscript and reviewed the manuscript for intellectual content. IS drafted the rest of the manuscript. All authors contributed to the article and approved the submitted version.

## Conflict of interest

IS was supported by a Grant Fondation Contre le Cancer (FCC-Post Doctoral funding; #2017-036), Fondation Saint-Luc for Cancer, Fondation Saint-Luc Unlock for Lives, and Fonds National pour la Recherche Scientifique (FNRS-CDR; #J.0161.21). The remaining author declares that the research was conducted in the absence of any commercial or financial relationships that could be construed as a potential conflict of interest.

## Publisher's note

All claims expressed in this article are solely those of the authors and do not necessarily represent those of their affiliated organizations, or those of the publisher, the editors and the reviewers. Any product that may be evaluated in this article, or claim that may be made by its manufacturer, is not guaranteed or endorsed by the publisher.
